# Dengue With COVID-19: Associated With Co-infection and Multiple Organ Dysfunction in a Child

**DOI:** 10.7759/cureus.20763

**Published:** 2021-12-27

**Authors:** Anima Ferdous, Mohammad Monir Hossain, Manifa Afrin, Mahfuza Shirin

**Affiliations:** 1 Pediatric Critical Care Medicine, Universal Medical College Hospital, Dhaka, BGD

**Keywords:** sars-cov-2, severe dengue, co-infection with dengue and covid-19, covid-19 in children, dengue with sars-cov-2

## Abstract

Coronavirus disease 2019 (COVID-19), due to SARS-CoV-2 infection, has been a global concern since January 2020. Southeast Asia, including Bangladesh, is facing outbreaks of endemic diseases such as dengue. Here, we report the case of an eight-year-old female from Dhaka with high-grade, continued fever, shock, features of pneumonia, and plasma leakage with multiple organ dysfunction. Both nonstructural protein 1 antigen (NS1 Ag) for dengue and reverse transcription-polymerase chain reaction (RT-PCR) for COVID-19 were positive in the patient.

The echocardiographic evaluation showed coronary arterial dilatations. The patient was managed according to the WHO guidelines for dengue with immunoglobulin, methylprednisolone, and aspirin for the involvement of coronary arteries. The patient required a mechanical ventilator due to pulmonary hemorrhage and unstable vitals. She showed gradual improvement with timely managements. Although a single case report does not portray the full picture, through this case report, we aim to describe the severity of co-infection of the mentioned viruses in a child in Bangladesh during the pandemic of SARS-CoV-2. Without appropriate diagnosis and management, it can be fatal.

## Introduction

Dengue fever, a mosquito-borne disease, is caused mainly by four serotypes of *Aedes aegypti*. Dengue became endemic in many regions, but Asia represents about 70% of the global burden [1,2]. Most cases of dengue are mild; however, due to an unpredictable clinical course, severe complications such as shock, hemorrhage, myocarditis, encephalopathy, and renal, hepatic, or multisystem impairments may develop in some cases [1,2].

During the monsoon season, Bangladesh is facing a double burden of a pandemic (SARS-CoV-2) and an endemic (dengue) simultaneously. More than 27,000 patients had been admitted to the hospital due to dengue since January 2021, while SARS-CoV-2 has affected more than 1,500,000 people since it was first detected until November 2021 [3,4]. This case report demonstrates the simultaneous infection of SARS-CoV-2 and severe dengue in a previously healthy child. Both diseases share some common clinical features and laboratory findings. Overlapping clinical and pathological similarities can lead to missing cases, which in turn can be fatal. A few cases regarding this co-infection are published, with rarity remaining in the pediatric age group [5]. Concomitant infection is likely to increase morbidity by several folds, especially in dengue-endemic countries.

## Case presentation

An eight-year-old, well-thriving female from Dhaka, weighing 34 kg, was admitted to the pediatric intensive care unit (PICU) with a four-day high-grade, continued fever and abdominal pain, diarrhea, and non-bilious vomiting, as well as a headache for the past three days. She has a history of contact with a family member with COVID-19 infection within a month of presenting illness. She had no previous history of dengue fever. On the second day of fever, nonstructural protein 1 antigen (NS1 Ag) for dengue was positive.

**Figure 1:**
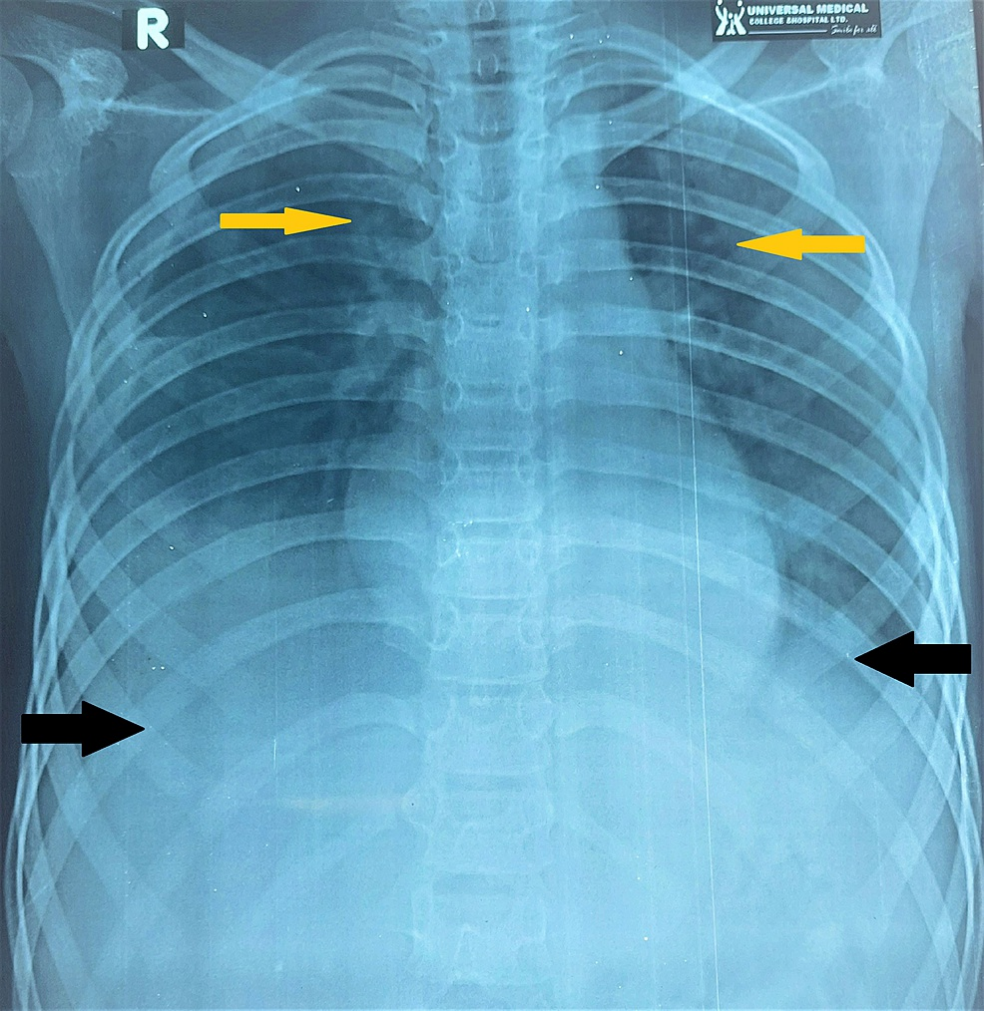
Portable chest radiograph anterior-posterior (A/P) view showing pleural effusion (black arrows) and pulmonary infiltrations (yellow arrows).

On examination, the patient was febrile, with a temperature of 102°F, and tachypnea (42/minute) and tachycardia (160/minute) were present, with low volume pulse and narrow pulse pressure of 60/45 mm Hg. Auscultation of lungs revealed coarse crepitations in both lungs with poor air entry, especially on the right middle and lower lung fields. The abdomen was distended with mild tenderness on the whole abdomen. Flanks were full, with no organomegaly. She had mucosal bleeding, petechial rashes on extremities, and generalized edema, with no lymphadenopathy. Oxygen saturation (SpO_2_) was 90% in room air and 96% with a face mask (5 L/minute oxygen). Initial investigations showed markedly reduced platelet counts and raised inflammatory markers. Serum calcium and albumin levels were low. Liver functions were altered with alteration of coagulation profiles (Table 1). Serum electrolytes and renal functions were within the normal range. The chest X-ray (CXR) revealed pulmonary infiltrations with pleural effusion (Figure 1).

**Table 1:** Investigation profile of the patient.

Investigations	Reference range	Before management	After management
Hemoglobin (g/dL)	Male: 12–17; female: 11.5–15.5	15.4	15.4
Hematocrit (%)	Male: 40–52; female: 36–48	35	36.9
Total white blood cell (WBC) (10^9^/L)	4–11	3.5	8.6
Neutrophils (%)	40–75	33	80
Platelet count (10^9^/L)	150–450	40	195
C-Reactive protein (mg/dL)	0.01–0.30	1.21	1.04
Serum procalcitonin (ng/mL)	<2.0	1.56	1.5
Serum albumin (g/dL)	3.4–5.0	1.8	5.06
Serum calcium (mg/dL)	8.20–10.20	6.0	8.9
Alanine transferase (ALT) (U/L)	<40	238	58
Aspartate aminotransferase (AST) (U/L)	<37	383	27
Prothrombin time (PT) (seconds)	Control: 12	16	12
Activated partial thromboplastin time (APTT) (seconds)	Control: 28	55	28
Blood urea (mg/dL)	10–40	40	20
Serum creatinine (mg/dL)	0.2–0.7	0.67	0.45
Serum ferritin (ng/mL)	7–140	>2000	209
D-dimer (mg/L)	<0.5	4.01	1.1
NT-pro-B-type natriuretic peptide (pg/mL)	<125	9432	759
Serum troponin I (ng/mL)	0.00–0.056	0.485	0.03

Blood, urine, stool, and throat swab culture were sent and came back with negative results. Nasopharyngeal swab for reverse transcription-polymerase chain reaction (RT-PCR) for SARS-CoV-2 was positive with a cycle threshold (CT) value of 32.9. The patient was isolated and treated for expanded dengue syndrome with plasma leakage with SARS-CoV-2 infection accordingly, with intravenous (IV) broad-spectrum antibiotics, inotropes, crystalloids, and colloids. Echocardiography on admission was normal with good biventricular systolic functions (ejection fraction (EF): 70%).

**Figure 2:**
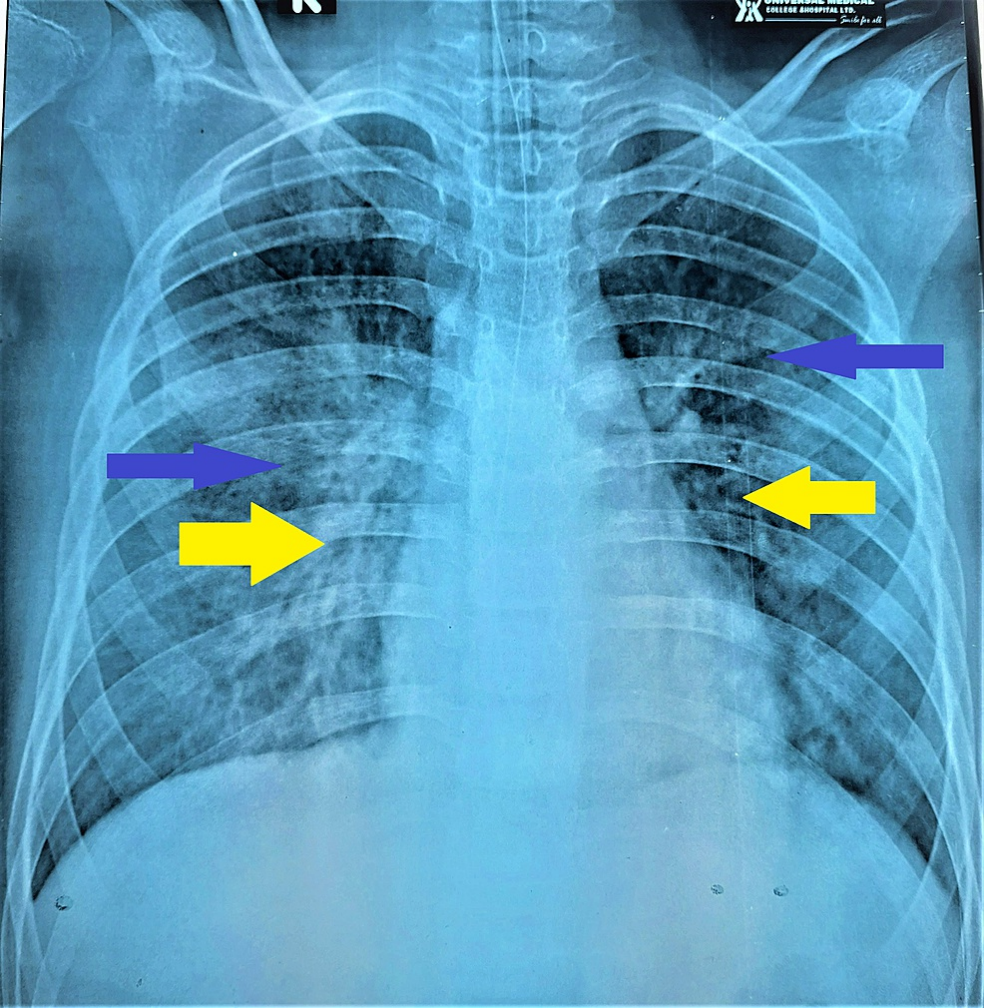
Chest X-ray of the patient during mechanical ventilatory support revealing bilateral alveolar opacities (blue arrows), suggesting pulmonary hemorrhage, and bilateral pulmonary infiltrations (yellow arrows).

Pleural effusion was resolving, but on the seventh day of fever, the patient developed a cough and increasing respiratory distress, with pulmonary hemorrhage. She was still febrile and hemodynamically unstable. Immediately, the patient was intubated and kept on a mechanical ventilator. Chest radiograph at that time revealed alveolar opacities and patchy infiltrations on both lungs (Figure 2).

**Figure 3:**
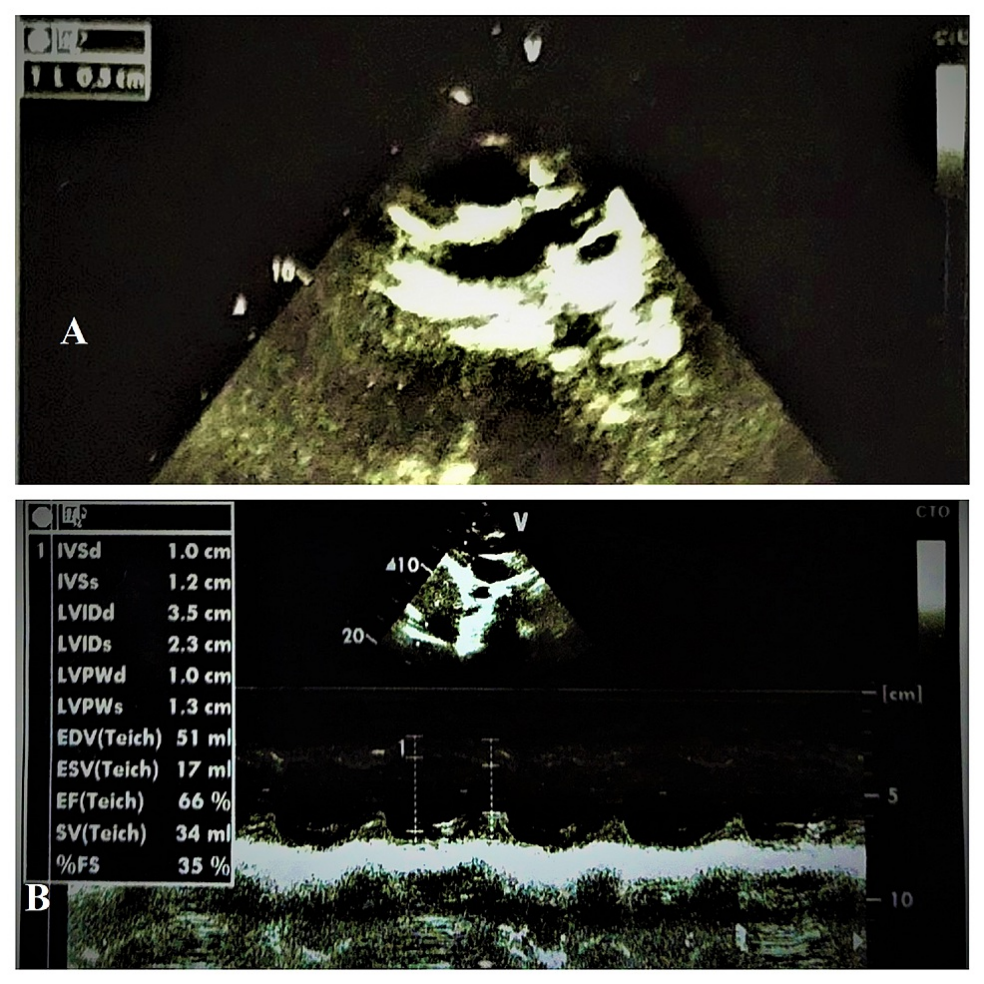
Echocardiograph of the patient showing dilated coronary artery (left main coronary artery (LMCA): 5 mm) (A) and good left ventricular systolic functions (ejection fraction: 66%) (B).

ECG was done and was still within normal limits. Dengue IgM was positive after seven days of fever. However, echocardiography on the seventh day revealed dilated coronary arteries (left main coronary artery (LMCA): 5 mm (+3 standard deviations (SD)); left coronary artery (LCA): 4.8 mm (+2.5 SD)), with the loss of distal tapering and perivascular brightness (Figure 3A) and fair biventricular systolic functions (EF: 66%) (Figure 3B).

Serum D-dimer and ferritin levels were noticeably increased with markedly raised cardiac enzymes (Table 1). Consultations were obtained from pediatric cardiologists, and we administered one dose of intravenous immunoglobulin (IVIG) 2 g/kg over 24 hours. Thereafter, methylprednisolone was added.

Meanwhile, the patient became afebrile and was hemodynamically stable with cessation of pulmonary hemorrhage. Chest radiographs showed improvements. Three days after ventilatory support, the patient was gradually extubated. General conditions were improving with normal vitals without inotropes, and SpO_2_ was 98% in room air with a gradual return of appetite. Inflammatory markers and cardiac enzymes were reduced. Hematocrit came to baseline with gradually corrected thrombocytopenia (Table 2) and coagulopathy. Aspirin was added subsequently.

**Table 2:** Hematocrit, total white blood cell (WBC), and platelet count during the course of disease of the patient.

Investigation	Reference value	First day of admission	Third day of admission	Seventh day of admission	Ninth day of admission
Hematocrit (%)	Male: 40–52; female: 36–48	35	46	27	36
Total WBC (10^9^/L)	4–11	3.5	6.2	9.4	8.6
Platelet count (10^9^/L)	150–450	40	21	62	120

At this point, the patient was transferred to the ward and thereafter discharged with the required and necessary advice and scheduled follow-ups.

## Discussion

SARS-CoV-2 infection in the pediatric age group is thought to cause less severe disease, but multiple organ dysfunction (multisystem inflammatory syndrome in children (MIS-C)) acquired from hyperinflammatory states and intensive care admission is a concern [6]. Feldstein et al. have shown that 73 (39%) of patients with MIS-C have positive RT-PCR for SARS-CoV-2 [7]. Both viruses, dengue and COVID-19, have many similar clinical features that overlap significantly and sometimes can be difficult to distinguish.

This case report describes coexisting severe dengue with COVID-19 in a previously healthy child with high-grade, continued fever, bleeding manifestations, respiratory distress, diffuse abdominal pain, emesis, and diarrhea. She was initially in profound shock with pleural effusion and pneumonitis. Patients with severe COVID-19 can present in such way, while severe dengue often gives these features. Pneumonic infiltrations on chest radiographs are consistent with SARS-CoV-2 infections [5-8]. Nevertheless, hemoconcentration and plasma leakage syndrome are the unique manifestations of severe dengue, which can help discriminate dengue from COVID-19 [8]. In dengue, abdominal symptoms and signs, such as pain, tenderness, and emesis, are commonly encountered. Diarrhea remains a common feature of COVID-19 infection [8]. Our patient required a mechanical ventilator for pulmonary hemorrhage (Figure 2) and unstable vitals, from which she recovered gradually. She did not have any palpable lymph nodes, strawberry tongue, or conjunctival congestion, commonly seen in Kawasaki disease. In severe dengue and severe COVID-19 infections or MIS-C due to COVID-19, plasma leakage with profound shock is not a very uncommon association [1,8,9].

Hematocrit rise of about 20% with concomitant lower platelet counts and leukocyte counts were observed in our patient, which settled to normal ranges with appropriate management (Table 2). Due to some common shared pathological pathways, both diseases can give features of plasma leakage, coagulopathies, and thrombocytopenia [8]. Although severe thrombocytopenia is uncommon with COVID-19, it is consistent with severe dengue [1,8]. In our patient, inflammatory markers were very high, with raised D-dimer level. Severe coagulopathy and altered liver functions were observed (Table 1). One of the main pathological associations in severe dengue is coagulopathy; however, it may also be seen in severe COVID-19 infection [1,8,9]. D-Dimer levels can be significantly high in both diseases. There is multiple evidence that suggests a hypercoagulable state in SARS-CoV-2 infection, which may lead to venous thromboembolism, pulmonary embolism, myocardial infarction, microvascular thrombosis, and even stroke [8,9]. Although in our patient, ECG was within normal limits, the cardiac enzymes were significantly raised (Table 1). During admission, the echocardiogram of the patient was normal, but subsequently, she developed coronary arterial dilatations (Figures 3A, 3B). A wide range of cardiac involvements, including sinus bradycardia, ST and T changes in ECG, raised cardiac enzymes, global hypokinesia, and ventricular dysfunction, are not uncommon with expanded dengue syndrome, with increased morbidity and mortality [1,2,10]. Coronary arterial involvement is commonly observed with Kawasaki disease or MIS-C due to SARS-CoV-2, but not evident in the case of dengue infection [1,2]. Few case reports suggested dengue infection might trigger Kawasaki disease [11]. Furthermore, raised troponin I, NT-pro-BNP, reduced ventricular functions, and abnormalities in coronary arteries can lead to adverse outcomes in severe COVID-19 infection or pediatric MIS-C [6,7].

Viral isolation and dengue serotype identification could not be done due to lack of facility.

## Conclusions

Concomitant severe dengue and SARS-CoV-2 could be associated with poor outcomes. The overlapping of the clinical and laboratory features of the mentioned diseases sometimes poses a challenge in accurate diagnosis and management. Point-of-care testing of COVID-19 should be sought in any febrile case during the pandemic, even if another infection has also been found. Isolation of febrile cases during COVID-19 is mandatory, but isolation may lead to delayed recognition and management of plasma leakage in severe dengue.
